# Combining Surgical and Percutaneous Revascularization for Multivessel Coronary Artery Disease: The Case for a Hybrid Approach

**DOI:** 10.1093/ejcts/ezaf406

**Published:** 2025-12-08

**Authors:** Samuel Heuts, Pim van der Harst, Erika Nodin, Joakim Grant Frederiksen, Patrique Segers, Fabrizio Rosati, Johan Bennett, Arpad Lux, Steven Jacobs, Monica Gianoli, Janus C Jakobsen, Christian Lildal Carranza, Wouter Oosterlinck

**Affiliations:** Department of Cardiothoracic Surgery, Maastricht University Medical Centre, Maastricht, The Netherlands; Cardiovascular Research Institute Maastricht, Maastricht University, Maastricht, The Netherlands; Department of Cardiology, University Medical Centre Utrecht, Utrecht, The Netherlands; Department of Cardiology, University Medical Centre Groningen, Groningen, The Netherlands; Department of Cardiothoracic Surgery, Rigshospitalet, Copenhagen, Denmark; Department of Cardiothoracic Surgery, Rigshospitalet, Copenhagen, Denmark; Department of Cardiothoracic Surgery, Maastricht University Medical Centre, Maastricht, The Netherlands; Division of Cardiac Surgery, ASST Spedali Civili di Brescia, Italy; Department of Cardiovascular Medicine, University Hospitals Leuven, Leuven, Belgium; Cardiovascular Research Institute Maastricht, Maastricht University, Maastricht, The Netherlands; Department of Cardiology, Maastricht University Medical Centre, Maastricht, The Netherlands; Department of Cardiac Surgery, University Hospitals Leuven, Leuven, Belgium; Department of Cardiothoracic Surgery, University Medical Centre Utrecht, Utrecht, The Netherlands; Copenhagen Trial Unit, Centre for Clinical Intervention Research, Rigshospitalet, Copenhagen University Hospital, Copenhagen, Denmark; Department of Regional Health Research, University of Southern Denmark, Denmark; Department of Cardiothoracic Surgery, Rigshospitalet, Copenhagen, Denmark; Department of Cardiac Surgery, University Hospitals Leuven, Leuven, Belgium

## INTRODUCTION

The burden of coronary artery disease (CAD) continues to rise, as a paradoxical consequence of improvements in medical therapy, an ageing population, and an increased prevalence of risk factors. Although optimal medical therapy (OMT) is the cornerstone of therapeutic strategies for CAD, (prompt) revascularization is indicated in patients with unstable disease, high-risk coronary anatomy, and ischaemic cardiomyopathy.^1^ Given the increasing risk profile of CAD patients, revascularization strategies may need to be tailored to each patient’s individual profile, balancing the trade-off between complete revascularization, periprocedural risk, and durability. This *Editorial* reviews the contemporary status of the combination of percutaneous and surgical revascularization options for multivessel CAD—a concept also known as “hybrid revascularization”—its underlying evidence base, conceptual considerations, and future directions.

## REVASCULARIZATION STRATEGIES

**Figure 1.**
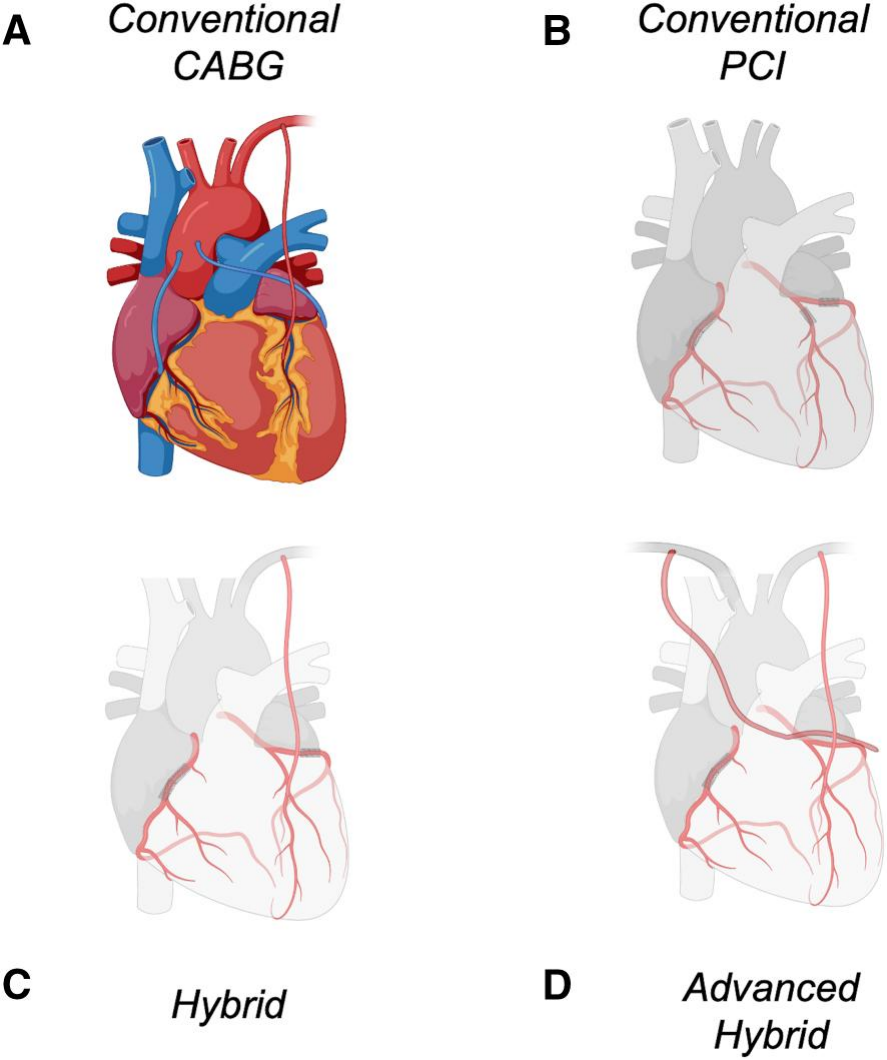
Modes of Revascularization. (A) Conventional CABG with LIMA-LAD and venous grafts to non-LAD targets, (B) multi-vessel PCI (LAD, Cx, RCA), (C) hybrid coronary revascularization; LIMA-LAD with multivessel PCI (Cx, RCA), and (D) advanced hybrid strategy during which both mammary arteries can be harvested through the same (left-sided) robotic working ports, and the grafting of the lateral wall can be achieved through a similar incision as for the LIMA-LAD anastomosis; MIDCAB LIMA-LAD, RIMA-Cx, and PCI of the RCA. Abbreviations: CABG: coronary artery bypass grafting; Cx: circumflex; HCR: hybrid coronary revascularization; LAD: left anterior descending; LIMA: left internal mammary artery; PCI: percutaneous coronary intervention; RCA: right coronary artery; RIMA: right IMA

Coronary revascularization can be achieved through 2 distinct modalities, each based on fundamentally different principles. First, a surgical strategy through coronary artery bypass grafting (CABG, **Figure 1A**) is aimed at the revascularization of ischaemic myocardial territories through the provision of a “surgical collateral”. This approach thereby treats the entire coronary artery, irrespective of calcific burden and presence of flow- or non-flow-limiting stenoses in the more proximal parts.^2^ The cornerstone of CABG is the grafting of the left anterior descending artery (LAD) with the left internal mammary artery (LIMA), providing an unequivocal survival and patency benefit as compared to the use of other grafts to the LAD or the grafting of non-LAD targets. The underlying explanation for this superiority arises from the combination of the large myocardial territory supplied by the LAD, and the physiological capabilities of the LIMA. The internal mammary arteries possess unique biological and haemodynamic properties that confer resistance to the development of atherosclerosis and subsequent late graft failure. Compared to other conduits for CABG, the mammary artery’s endothelium shows enhanced nitric oxide synthesis, which benefits not only the graft but also the downstream coronary circulation, while it also exhibits flow-mediated vasodilation. In addition, the mammary artery shows more elastic (and less muscular) properties than, for example, the radial artery, rendering the IMA less prone to vasospasm or competitive flow. Thus, CABG is particularly indicated for patients with at least single-vessel LAD-disease, and usually performed in patients with double- and triple-vessel disease, with or without involvement of the left main coronary artery. The clinical long-term benefit of CABG, as compared to percutaneous interventions, is also well established and derived from large multicentre randomized controlled trials, such as the *Synergy between PCI with Taxus and Cardiac Surgery* trial (SYNTAX).^3^ In the 5-year follow-up of major adverse cardiovascular events (all-cause mortality, stroke, myocardial infarction (MI), and repeat revascularization), there was a significant benefit of CABG (26.9%) as compared to percutaneous coronary intervention (PCI) (37.3%, *P* < .001).^3^ This difference was particularly apparent in patients with high-complexity CAD, expressed in the SYNTAX-score (for which a score <22 indicates low-complexity CAD).^3^ Moreover, in the extended 10-year follow-up survival analysis, all-cause death was significantly increased in patients undergoing PCI (28% as compared to 21% with CABG) with 3-vessel disease. In addition to high-complexity CAD, CABG also performs better in patients with a more extensive risk profile, such as those with diabetes or ischaemic cardiomyopathy. Indeed, both the *Future Revascularization Evaluation in Patients with Diabetes Mellitus: Optimal Management of Multivessel Disease* (FREEDOM) and *Surgical Treatment for* Ischaemic *Heart Failure—Extended Survival* (STICHES) trials demonstrated improved survival in patients undergoing CABG as compared to PCI (FREEDOM) or medical therapy (STICHES).^4^^,^^5^ Based on these landmark trials, current guidelines recommend CABG over PCI in patients with multivessel disease, particularly in the presence of high-complexity CAD as reflected by the SYNTAX-score, diabetes, and/or a markedly reduced ejection fraction.^1^^,^^6^

Nevertheless, CABG is an invasive procedure, mostly requiring access through sternotomy, cardiopulmonary bypass (CPB), and use of cardioplegic arrest—all of which are associated with a non-negligible risk of stroke and periprocedural morbidity and mortality. Although techniques such as off-pump CABG (OPCAB) avoid aortic manipulation, their adoption is relatively limited due to increased technical complexity and heterogeneity in outcomes, as shown in RCTs where conflicting results have been reported in terms of incomplete revascularization, periprocedural MI and survival.^7^ In general, CABG can be considered across the spectrum of CAD (ie, chronic and acute) to optimize prognosis, although it is less applicable as an emergency procedure for ST-elevation MI (STEMI).^1^

PCI (**Figure 1B**) provides a less invasive revascularization option. PCI differs in its conceptual treatment of CAD and addresses functionally flow-limiting focal stenoses in chronic and acute coronary syndromes (CCS/ACS), or culprit lesions during ACS. Several innovations have improved PCI therapy, including the use of the latest generation drug-eluting stents (DES, lowering restenosis risk), functional assessment (through use of fractional flow reserve, aimed at the objective identification of haemodynamically significant obstructions), and imaging-guided treatment decisions (intravascular ultrasound or optical coherence tomography, optimizing stent sizing and evaluation). Currently, PCI is the unequivocal first-line treatment for STEMI—optimally preserving viable myocardium in emergency situations. Furthermore, PCI is used to treat non-STEMI (NSTEMI), unstable angina, and CCS, although the prognostic benefit in the latter patient population is less clear.

Nevertheless, both therapies can also “fail,” in the early and late phase, though through different mechanisms. Mechanisms of *early* bypass graft failure comprise technical causes, such as anastomotic occlusions, conduit kinking, or thrombosis. Then, arterial grafts in general, and radial artery grafts in particularly, are prone to spasm (string-sign) in presence of native coronary artery competitive flow. Consequently, radial grafts should preferably be used in the presence of critical (>90%) stenoses. Over the longer term, vein grafts are especially susceptible to neo-intimal thickening (intimal hyperplasia) and development of de novo atherosclerotic disease, which is characterized by the presence of multiple friable, lipid-rich plaques that lead to vein graft stenosis or occlusion. On the other hand, stent failure also has a multifactorial underlying aetiology, albeit different from bypass graft failure. In the early phase, procedural factors, such as stent underexpansion, edge dissection, malapposition, or acute thrombosis may result in the devastating event of a (sub)acute stent occlusion. Then, although the rate of in-stent restenosis has markedly declined with the introduction of (second-generation) DES, it remains a relevant complication in a subset of patients, particularly those with diabetes and small-calibre vessels. Finally, as PCI is a focal therapy of a flow-limiting stenosis, it does not protect the vessel from future MI secondary to non-stented unstable plaques. Consequently, PCI is associated with higher rates of target vessel revascularization as compared to CABG.

Although several randomized controlled trials and subsequent meta-analyses^3–5^^,^^8^ have established the superiority of CABG over PCI in patients with complex CAD, it remains unclear whether this benefit is primarily determined by the LIMA-LAD graft. As such, the combination of a (minimally invasive) LIMA-LAD procedure with multivessel PCI of non-LAD targets, known as hybrid coronary revascularization (HCR), could be an attractive alternative to suitable patients. Nevertheless, this combination of both procedures in a hybrid setting is less well studied in literature.

## COMBINING THE BEST OF BOTH WORLDS: HYBRID REVASCULARIZATION

The combination of CABG LIMA-LAD and (staged) non-LAD PCI into one procedure is termed “hybrid coronary revascularization” (HCR). It involves incorporating the gold standard of CABG (the LIMA-LAD anastomosis) into a multivessel PCI strategy for non-LAD targets,^9^ in carefully selected patients. Given the location of the LAD in the anterior myocardial wall, the surgical part of this procedure does not require sternotomy, as the anastomosis can be performed through an anterolateral left mini-thoracotomy (minimally invasive direct coronary artery bypass grafting [MIDCAB]). Moreover, systemic impact is diminished by the use of the off-pump technique, which also reduces the risk of stroke. In addition, surgical trauma can be minimized even further through the use of a video-assisted or robotic-assisted approach (RA-MIDCAB), in which the LIMA can be harvested through port incisions. The first case of a MIDCAB approach combined with PCI was reported as early as 1996. Still, its widespread adoption has been limited.

In the hybrid approach, following the surgical MIDCAB procedure, complete revascularization for multivessel disease can be achieved through PCI of the non-LAD target(s) (**Figure 1C**). Most often, this is realized through a staged procedure, which occurs days to weeks following the index operation. In this setting (MIDCAB before PCI), the need for dual antiplatelet treatment (DAPT) is obviated during MIDCAB, while facilitating verification of the LIMA-LAD bypass when invasive coronary angiography is performed at the time of PCI. Still, in case of unstable symptoms or ACS in which the culprit comprises a non-LAD vessel, PCI may be performed prior to MIDCAB—known as a “reversed hybrid procedure.” If possible, MIDCAB can be postponed to 6 weeks to 3 months following latest generation DES-PCI implantation, when temporary discontinuation of DAPT may be considered. Alternatively, in a “reversed hybrid” approach where more prompt revascularization of the LAD is indicated, the MIDCAB procedure can be performed under DAPT, albeit at a higher risk of bleeding.

This balance between thrombotic and bleeding risk is more evident in patients undergoing HCR than conventional CABG, as more aggressive antiplatelet therapy is warranted due to the associated PCI and subsequent risk of stent thrombosis. Most patients are not at high bleeding risk, but such considerations may be even more important in patients with indications for concomitant oral anticoagulation, such as atrial fibrillation (AF) or venous thrombo-embolisms. Although triple therapy (DAPT with oral anticoagulation) is discouraged in current guidelines,^6^ the combination of a (direct) oral anticoagulant with a P2Y12-inhibitor (such as clopidogrel) carries a high bleeding risk as well. Despite the fact that a MIDCAB procedure can be combined with thoracoscopic surgical ablation for AF with concomitant closure of the left atrial appendage, the heart-team must strongly consider the benefit of HCR over conventional CABG in high bleeding risk patients with an indication for anticoagulation.

Finally, the benefit of arterial grafts may be most evident when the left coronary system is grafted. Therefore, with increasing surgical proficiency in RA-MIDCAB and a focus on achieving more complete surgical arterial revascularization, the hybrid approach may even evolve into an “advanced hybrid strategy” (**Figure 1D**), combining full arterial revascularization of the left coronary system with DES-PCI of the right coronary artery. In this approach, both mammary arteries can be harvested through the same (left-sided) robotic working ports, and the grafting of the lateral wall can be achieved through a similar incision as for the LIMA-LAD anastomosis. Although seemingly promising, it must be mentioned that the results of this latter strategy are not yet widely reported.

## THE EVIDENCE BASE FOR A HYBRID APPROACH

Despite its introduction almost 3 decades ago, the evidence base for a hybrid approach remains limited. To date, only 3 RCTs have been published.^10–12^  **Table 1** presents their most pertinent findings. Across these RCTs, no statistically significant differences were observed between HCR and conventional CABG (or multivessel PCI) in terms of major adverse cardio- and cerebrovascular events (MACCE), early mortality or long-term survival, or repeat revascularization during follow-up. Indeed, 5-year outcomes in Tajstra et al^11^ (POL-MIDES), 3-year results from Ganyukov et al^10^ (HREVS), and 2-year results from Esteves et al^12^ (MERGING) all demonstrated comparable survival and MACCE rates between groups. The 3 trials collectively suggest that HCR offers similar mid-term clinical efficacy to CABG (or PCI), with acceptable procedural safety. Of note, these trials reported on clinical end-points and did not incorporate standardized angiographic follow-up to evaluate graft patency. Still, recurrent limitations among all of the 3 trials are a relatively small sample size with a limited follow-up, potentially underestimating clinically relevant differences between groups. In addition, there was “some concerns” of risk of bias for all 3 trials, using the Risk of Bias (RoB) 2.0 tool as advocated by the Cochrane Collaboration, emphasizing the lack of high quality randomized data in this area, which should preferably be derived from multicentre initiatives such as is the case for conventional CABG.^3–5^

**Table 1.** The Randomized Evidence Base for HCR

	Published trials						
First author	Year of publication	Number of patients	Trial arms	Risk of bias assessment^a^	Follow-up	End-points	Main findings
Tajstra et al^11^	2018	200 patients with MVCAD and CABG indication	HCR (*n* = 102) vs CABG/OPCAB (*n* = 98)	Some concerns	Median follow-up of 5.9 years	5-year all-cause mortality and MACCE	5-year mortality 6.4% (HCR) vs 9.2% (CABG, *P* = .69) without differences in MACCE (*P* = .39) or reinterventions (*P* = .38)
Ganyukov et al^10^	2021	155 patients with MVCAD	HCR (*n* = 52) vs CABG (*n* = 50) vs PCI (*n* = 53)	Some concerns	Mean follow-up of 52 months (and at least 3 years)	All-cause mortality, MACCE, TVR	3-year mortality 6.3% (HCR) vs 4.0% (CABG) vs 6.0% (PCI, *P* = .89) with no differences in MACCE (*P* = .18) or TVR (*P* = .87)
Esteves et al^12^	2020	60 patients with complex 3VD in a 2:1 ratio	HCR (*n* = 40) vs CABG (*n* = 20)	Some concerns	Mean follow-up of 802 days	All-cause mortality and MACCE	MACCE 19.3% (HCR) vs 5.9% (CABG, *P* = .2), mortality 5.0% (HCR) vs 0 (CABG)

a Using the RoB 2.0 tool as advocated by the Cochrane Collaboration.

Abbreviations: CABG: coronary artery bypass grafting; HCR: hybrid coronary revascularization; MACCE: major adverse cardio- and cerebrovascular events; MVCAD: multivessel coronary artery disease; OPCAB: off-pump CABG; PCI: percutaneous coronary intervention; TVR: target vessel revascularization.

Nevertheless, several non-randomized multicentre observational cohort studies have demonstrated the safety and feasibility of a hybrid approach.^13^ Although comparative analyses derived from non-randomized samples are less appropriate, Puskas et al^13^ found that a hybrid approach was associated with excellent survival outcomes and low rates of repeat revascularization at maximum follow-up. Particularly the multicentre nature of this study emphasizes the generalizability of the procedure.

Given the paucity of high-quality RCTs, current European guidelines only provide a class IIb indication (*could be considered—*level of evidence B) for hybrid revascularization in specific subsets of CAD patients at experienced centres.^1^

The limited adoption of the hybrid approach may be partially explained by this lack of high-quality evidence demonstrating its clinical benefit, though other factors may play a role as well. First, HCR requires close collaboration between surgeons and cardiologists, often transcending traditional professional boundaries, and its adoption therefore heavily depends on the maturity of the local heart-team. Furthermore, particularly the surgical part of the procedure (MIDCAB LIMA-LAD, often performed with robotic assistance) is associated with a marked learning curve, potentially deterring surgeons from being invested in this procedure. Also, most countries lack specific reimbursement for HCR, despite the significant costs of 2 separate procedures, often requiring 2 hospitalizations—which may be, hypothetically, offset in the long-term due to a lower complication rate and less (coronary) reinterventions.

## CONCEPTUAL CONSIDERATIONS

As no adequately powered RCT has been conducted yet that is able to detect potential differences in hard clinical end-points, the current understanding of HCR’s clinical efficacy must, for now, be derived indirectly from studies on PCI and CABG.

The LIMA-LAD graft has proven to be superior to all other modalities of revascularization to the LAD (ie, other grafts or PCI), with long-term patency rates reported of over 95%.^14^ This intuitively sets the stage for the surgical part of the MIDCAB LIMA-LAD procedure during a hybrid approach.

Moreover, it has been widely recognized that complete revascularization improves outcomes as compared to *incomplete* revascularization. Still, the severity and complexity of CAD—in combination with calcific burden, which sometimes precludes complete revascularization—are obvious confounders in this comparison. Indeed, patients with more extensive CAD may be exposed to less complete revascularization, but their increased risk profile and atherosclerotic burden may primarily determine prognosis, regardless of completeness of revascularization. It must be mentioned that the definition of “complete revascularization” varies markedly in the literature as well.

In a recent registry-based analysis, there was no longer a difference between PCI and CABG when controlling for completeness of revascularization.^15^ Still, the completeness of revascularization rate was lower with PCI than CABG in this study (20% vs 93%), implying this equivalence in outcomes can only be achieved 1 in 5 PCI cases. Consequently, the indication for a hybrid approach must not only take the patient’s risk profile into account, but also the likelihood of complete surgical LAD revascularization and percutaneous revascularization of non-LAD targets.

Indeed, the evidence for prognostic superiority of the surgical grafting of the LAD (by the LIMA) is undisputable, with consistent reduction in mortality and MI, due to a combination of the long-term patency of the LIMA-LAD graft and the large amount of myocardium supplied by the LAD. However, such prognostic benefit is less well established for the surgical grafting of non-LAD targets versus PCI of such vessels. It remains difficult to directly compare the patency rates and subsequent impact of PCI and non-LIMA grafting of non-LAD targets. The long-term patency rate of vein grafts, radial arteries, and the RIMA varies between 75% and 93%, depending on the target vessel,^14^ and can be as low as 50% after 10 years. Although non-comparative, contemporary DES-PCI registries have demonstrated clinically significant stent-restenosis rates of <5% at 2 years, suggesting potential equivalence between PCI and non-LIMA grafts for non-LAD targets.

Nevertheless, revascularization is not only performed for prognostic benefit, but also for improvement in quality of life and reduction of angina and exertional dyspnoea. Indeed, CABG’s ability to improve angina is well established and therefore also specifically stated as an indication with class I recommendation in contemporary guidelines.^6^ Unfortunately, there is a scarcity of data on these important patient-reported outcomes in HCR literature.

All in all, hybrid revascularization may not be ideal for all patients, particularly those with diffuse CAD in non-LAD vessels not suitable for PCI, or those who are at increased risk of incomplete revascularization. Conversely, patients with a combination of a (complex) proximal or diffuse LAD lesion and focal lesions in non-LAD targets (or surgically unfavourable targets) seem particularly amenable to this approach. Moreover, patients with increased surgical risk, contraindications to aortic manipulation, and/or decreased suitability for sternotomy, seem to ideally qualify for this less invasive approach. Together, these features minimize surgical trauma while increasing the likelihood of complete revascularization, thereby optimizing outcomes in this selected patient population. **Table 2** therefore summarizes the ideal features to establish an indication for HCR, alongside absolute and relative contraindications for this procedure, including coronary anatomical aspects, comorbidities and clinical factors, and surgical features. Although the definitiveness of absolute and relative contraindications may differ between institutions and surgeons, and may change following the accumulation of more expertise, the currently listed features particularly apply to the general HCR patient in a centre with considerable expertise in MIDCAB and HCR.

**Table 2.** Patient Selection for Hybrid Coronary Revascularization

Feature	Favourable features	Absolute and relative contraindications
Coronary anatomy		
LAD	The majority of LAD disease is suitable for LIMA-LAD grafting, though surgical grafting particularly excels, as compared to PCI, in complex proximal LAD lesions and diffuse LAD disease	Chronic total occlusion without collateral filling and/or myocardial viability
Non-LAD vessels	Focal non-LAD lesions with demonstrated haemodynamic consequences (ie, FFR ≤0.80 and/or RFR/iFR ≤0.89) High likelihood of complete revascularization with PCI	Diffuse non-LAD disease Small (<2 mm) vessels Chronic total non-LAD occlusions Severely calcified vessels requiring calcium-modification therapies
Comorbidities and clinical factors	Although patients at low surgical risk could also be ideal candidates for HCR, this procedure may be particularly applicable in patients at elevated surgical risk (ie, EuroSCORE II >2%) Elective and urgent (potentially less-favourable) surgical indications in stable patients	Inability to tolerate DAPT due to (very) high bleeding risk, particularly in combination with the requirement for (D)OACs Severe (chronic) pulmonary disease prohibiting the use of single-lung ventilation Haemodynamically unstable patients and those with an emergency indication for revascularization (ongoing ischaemia/infarction) Other indications for concomitant cardiac surgery such as moderate-severe valve disease
Surgical factors	Suitable thoracic anatomy for a left-sided MIDCAB approach	Severe thoracic deformities, such as (severe) pectus excavatum Prior left thoracotomy Prior thoracic radiation therapy
Other	Other reasons that mandate the avoidance of sternotomy Explicit patient preference Experienced hybrid team and MIDCAB-surgeons	Limited institutional logistics

Abbreviations: DAPT: dual antiplatelet therapy; (D)OAC: (direct) oral anticoagulant; FFR: fractional flow reserve; HCR: hybrid coronary revascularization; iFR: instantaneous wave-free ratio; LAD: left anterior descending artery; LIMA: left internal mammary artery; MIDCAB: minimally invasive direct coronary artery bypass grafting; PCI: percutaneous coronary intervention; RFR: resting full-cycle ratio.

## FUTURE DIRECTIONS

Despite proven feasibility in experienced surgical centres, the current evidence base is too limited to recommend a hybrid strategy to all-comer patients with complex CAD in all surgical centres. Adequately powered trials are highly warranted to compare the net benefit of HCR versus conventional multivessel CABG. Also, an expansion of the indication for HCR may paradoxically increase the number of surgical LIMA-LAD candidates, as patients previously offered multivessel PCI may become eligible for HCR. Furthermore, HCR may have a meaningful impact beyond conventional clinical end-points, particularly on patient quality of life. By avoiding sternotomy and CPB, HCR can reduce postoperative pain, facilitate faster mobilization and functional recovery, preserve independence, while improving overall patient satisfaction. In addition, the approach may yield relevant societal and system-level benefits. The avoidance of sternotomy can shorten hospitalization and intensive care stay, which is cost-saving and enables higher surgical throughput—an important consideration given the increasing strain on healthcare systems, rising costs, an ageing population, and limited bed capacity. Moreover, an expedited recovery and rehabilitation can allow patients to resume normal activities and work sooner, further reducing societal expenditure. Therefore, beyond traditional hard end-points, patient-reported outcome measures, length of hospital stay, cost-effectiveness, and comprehensive health-economic evaluations could be integrated into future trials assessing the full value of HCR.

**Table 3** presents currently planned trials, as listed in trial registries. Although the NHLBI-funded HYBRID trial (NCT03089398) was adequately powered to detect differences in MACCE between HCR and PCI, the trial was prematurely discontinued due to slow enrolment. Therefore, the upcoming European trial (NCT05504031, HYCORE1) comparing HCR to CABG seems especially promising. With a planned sample size of 1048 patients, the trial is powered to detect an absolute risk difference of 10% in MACCE or unplanned rehospitalization within 12 months.

**Table 3.** Recently Registered Ongoing or Completed Trials on HCR That Have Not (Yet) Been Published

Registered trials						
NCT registration	Year of registration	Prospected sample size	Trial arms	Prospected follow-up	End-points	Estimated year of completion
NCT03089398	2017	2354	Multivessel PCI vs HCR	24 months	MACCE, LOHS, cost-effectiveness	2021—eventually discontinued for slow enrolment
NCT04811586	2021	200	One-staged HCR vs PCI	24 months	MACCE and its individual components	Unknown status
NCT05504031	2022	1048	HCR vs CABG	12 months	MACCE and unplanned rehospitalization at 12 months	Enrolment still to start
NCT06378775	2024	80	RA-MIDCAB HCR vs CABG	30 days	LOHS, conversion, ICU stay	Enrolment still to start

Abbreviations: CABG: coronary artery bypass grafting; HCR: hybrid coronary revascularization; LOHS: length of hospital stay; PCI: percutaneous coronary intervention; MACCE: major adverse cardio- and cerebrovascular events; NCT: national clinical trial; NR: not reported.

Based on the findings of large RCTs, the recommendation for HCR may be modified in future guidelines, while the indication for HCR may be expanded to patients with a variety of surgical risk—including those at a young age—under the condition of the fulfilment of coronary anatomical and surgical criteria. Given the importance of multidisciplinary collaboration and patient selection, the heart-team is paramount to the future of HCR.

## CONCLUSION

HCR combines the prognostic benefit of the surgical LIMA-LAD graft with the reduced invasiveness offered by PCI for non-LAD lesions. Nevertheless, HCR’s current evidence base remains weak, and only supports this combined procedure to be performed in expert centres, in selected patients. Careful patient selection is imperative, particularly with regard to the likelihood of complete revascularization achievable with PCI of non-LAD targets. Large and adequately powered trials are urgently needed to definitely establish HCR’s role across the revascularization spectrum.

## Data Availability

No new data were generated for this Editorial.
